# An Overlooked Factor in the Appeal for the Blind

**Published:** 1914-05-30

**Authors:** C. Waring

**Affiliations:** Hon. Sec. London Spectacle Mission, 197 Sutherland Avenue, W.


*To the Editor of* The Hospital.

### An Overlooked Factor in the Appeal for the Blind.

*To the Editor of* The Hospital.

Sir,—At this moment, when such a splendid movement is afoot as that for assisting the blind, may I put in a plea for a kindred object, which affects a much larger number of people? I mean spectacles for those poor and hard-working folk whose sight is failing from age and infirmity.

While the blind number thousands, those with failing sight number tens of thousands.

The London Spectacle Mission aims at supplying these sufferers on life's journey with suitable, strong, and well-made spectacles, and in the last twenty years some 40,000 people have been so supplied.

All money given entitles the donor to recommend applicants for this Charity, which has centres in five different parts of London—North, South, East, West—and the head centre at 197 Sutherland Avenue, W.

All particulars will be gladly given on application to the Hon. Secretary.—Yours faithfully,

C. Waring, Hon. Sec.

London Spectacle Mission,
197 Sutherland Avenue, W.

[Note.—*Owing to the pressure on our space we have had to hold over several letters this week.*]

